# Identification and clinical evaluation of segments of homozygosity, uniparental disomy and complex chromosomal abnormalities revealed by copy-number SNP arrays

**DOI:** 10.1186/1755-8166-7-S1-O4

**Published:** 2014-01-21

**Authors:** Jia-Chi Wang, Leslie Ross, Loretta W Mahon, Renius Owen, Morteza Hemmat, Boris T Wang, Mohammed El Naggar, Kimberly A Kopita, Mary Haddadin, Fatih Z Boyar, Arturo Anguiano, Charles M Strom, Trilochan Sahoo

**Affiliations:** 1 Cytogenetics Laboratory, Quest Diagnostics Nichols Institute, 33608 Ortega Highway, San Juan Capistrano, CA, USA

## Background

Presence of such segments of homozygosity (SOH) may be due to parental relatedness, chromosomal recombination or rearrangements and provides important clues regarding ancestral homozygosity, parental consanguinity or uniparental disomy. We have determined the frequency and nature of copy neutral segments with allelic homozygosity identified in cases interrogated by oligonucleotide-SNP microarrays.

## Materials and methods

We collected cases from consecutive specimens sent to our clinical laboratory over the past two years. The cases were reported based on the presence of a contiguous SOH >10 Mb in a single region or >5 Mb in at least two regions. The percentage of the genome encompassed by SOH regions was calculated based on the total coverage of about 2,700 Mb.

## Results

Of 14,574 cases analyzed by SNP arrays, 872 (6%) cases harbored SOH, with 659 (76%) cases harboring multiple SOH and interpreted as arising due to identity by descent (IBD), 213 (24%) cases with SOH involving a single chromosomal segment and suspected or confirmed as resulting from UPD. For the cases with IBD, the coefficient of inbreeding was calculated: 5% cases due to first degree or closer parental relatedness, 9% second, 19% third, 16% fourth, and 51% fifth. Cases with UPD cases involved every single chromosome. In eight cases, identification of SOH was crucial to diagnosing autosomal recessive disorders.

## Conclusions

This study demonstrates that the identification of SOH, in addition to CNVs, is much more frequent than previously recognized, reflecting close parental relatedness, and often ascertains autosomal recessive diseases or unravels UPD in many cases.

